# Wavelet-Based Artifact Identification and Separation Technique for EEG Signals during Galvanic Vestibular Stimulation

**DOI:** 10.1155/2013/167069

**Published:** 2013-09-01

**Authors:** Mani Adib, Edmond Cretu

**Affiliations:** Department of Electrical and Computer Engineering, The University of British Columbia, Vancouver, BC, Canada V6T 1Z4

## Abstract

We present a new method for removing artifacts in electroencephalography (EEG) records during Galvanic Vestibular Stimulation (GVS). The main challenge in exploiting GVS is to understand how the stimulus acts as an input to brain. We used EEG to monitor the brain and elicit the GVS reflexes. However, GVS current distribution throughout the scalp generates an artifact on EEG signals. We need to eliminate this artifact to be able to analyze the EEG signals during GVS. We propose a novel method to estimate the contribution of the GVS current in the EEG signals at each electrode by combining time-series regression methods with wavelet decomposition methods. We use wavelet transform to project the recorded EEG signal into various frequency bands and then estimate the GVS current distribution in each frequency band. The proposed method was optimized using simulated signals, and its performance was compared to well-accepted artifact removal methods such as ICA-based methods and adaptive filters. The results show that the proposed method has better performance in removing GVS artifacts, compared to the others. Using the proposed method, a higher signal to artifact ratio of −1.625 dB was achieved, which outperformed other methods such as ICA-based methods, regression methods, and adaptive filters.

## 1. Introduction

**Figure 1:**
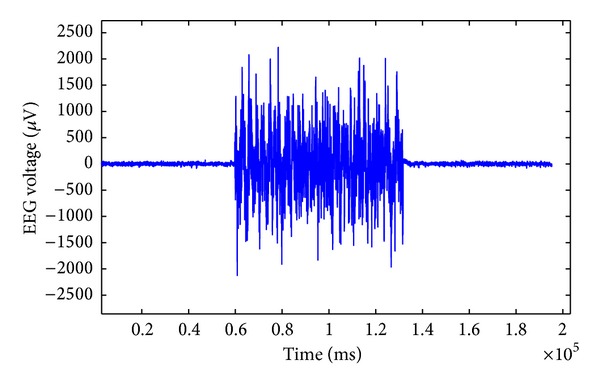
Measured EEG data during 72 seconds of GVS stimulation and 60 seconds before and after applying the GVS.

Brain stimulation by means of electrical currents has been employed in neurological studies for therapy purposes for many years [1–5]. However, the ability to analyze the ongoing neural activities during the stimulation is limited due to the artifact generated by GVS. The leakage of the stimulation current through the scalp generates an additional electrical potential with a much higher amplitude than that of the neural activities. As a result, higher artifactual potentials are collected by the EEG electrodes, especially in the neighbourhood of stimulation areas. The stimulation artifacts which are superimposed on the EEG signals are the main obstacle in understanding the effects of the GVS interactions with neural circuitries in different brain regions. Analyzing the EEG signals during GVS stimulation is of high importance, as it provides information on how it affects the neural activities. For instance, in suppressing the symptoms of some neurological disorders using GVS, researchers are interested in eliciting GVS responses in different brain regions. Furthermore, to be able to perform GVS studies in closed-loop mode, where the delivered GVS stimuli are adjusted in response to ongoing neural activities, it is necessary to remove the stimulation artifacts from neural activities signals. An experimentally measured example of EEG signals contaminated with the GVS artifacts is illustrated in Figure 1.

 Considering that the frequency spectra of the neural signals and GVS artifacts overlap, filtering the frequency components of GVS artifacts results in the loss of the original neural signals. The four major EEG frequency bands are Delta (the lowest frequency band up to 4 Hz), Theta (4 Hz to 8 Hz), Alpha (8 Hz to 12 Hz), and Beta (12 Hz to 30 Hz). In order to analyze and understand the effect of GVS on EEG patterns, it is essential to be able to remove the artifact signals from the frequency band of interest, before establishing any GVS-EEG interaction models.

There are various methods to remove different types of artifacts, such as myogenic artifacts [6–9], ocular artifacts [10–15], extrinsic artifacts such as MRI induced artifacts in simultaneous EEG/fMRI studies [16], stimulation artifacts [17–20], and general artifacts and signals that have noncerebral origin [21, 22]. One of the most commonly used methods to remove artifacts from EEG signals is the Independent Component Analysis (ICA). Generally, in the component-based methods such as ICA, the EEG signals are decomposed into statistically independent and uncorrelated terms; the artifact components are then identified and filtered out, and the EEG signals can be reconstructed from the neural components without artifacts. However, applying ICA to remove the GVS stimulation artifacts is challenging, particularly when we increase the amplitude of the GVS over 1 mA with a signal to artifact ratio less than −35 dB. We will discuss this in more detail later in the section “Comparison of the performance of different artifact removal methods”.

We propose a novel method for GVS artifacts removal by combining time-series regression methods and wavelet decomposition methods. To enhance the precision of the artifact estimation using regression models, the models should account for the complex behavior of the GVS interactions in the frequency domain. So we decomposed the recorded EEG and GVS signals into different frequency bands and then used regression models to estimate the GVS artifacts in each frequency band. We used multiresolution wavelet analysis to decompose nonstationary EEG signals in the time-frequency plane. Both the discrete wavelet transform (DWT) and the stationary wavelet transform (SWT) algorithms were employed, and the results were compared. To estimate the GVS current distribution through the scalp using time-series regression methods based on biophysical models, we used and compared the performance of different parametric regression models, such as discrete-time polynomials, nonlinear Hammerstein-Wiener, and state-space models.

In this study, we firstly used simulated data to assess and optimize the performance of the proposed method using various regression models and different wavelet algorithms. The resulting optimized method was then applied to real data. We compared the results of the proposed method and other methods, such as ICA, using both simulated and real data. This paper is organized as follows: Section 2 provides a detailed description of the equipment and set-up, the data simulation, the signal processing methods, and the comparison of their performances.  Section 3 shows the results of the proposed artifact removal method, and in Section 4, we discuss the proposed method, its results, and suggested works for the future.

## 2. Materials and Methods

### 2.1. Equipment and Setup

 The EEG recording was carried out with a NeuroScan SynAmps2 system, with 20 electrodes located according to the international 10–20 EEG system (Table 1) and with a sampling frequency set to 1 kHz.

 The GVS signal was applied using a Digitimer DS5 isolated bipolar current stimulator. This stimulator can generate a stimulation current with a waveform proportional to the controlling voltage applied to its input. The waveform was generated using LabVIEW and sent to the stimulator through a National Instrument (NI) Data Acquisition (DAQ) board. In this study, we applied a zero-mean pink noise current, with a 1/*f*-type power spectrum within a frequency range of 0.1 to 10 Hz and duration of 72 seconds. We kept the amplitude of the delivered stimuli lower than the feeling threshold, in the range of 100 *μ*A to 800 *μ*A, with the root mean square values between 60 *μ*A and 450 *μ*A. The stimulator is equipped with a data acquisition device to record the delivered stimulus, which allows us to make a continuous record of the delivered stimulation current and voltage. We recorded the EEG signals during the stimulation, 60 seconds before and 60 seconds after the stimulation. The EEG data for these experiments were acquired by our collaborator in the Pacific Parkinson's Research Centre. Nine healthy subjects (6 males, 3 females), between the ages of 21 and 53 yr, with no known history of neurological disease or injury, participated in this study. All subjects were asked to relax, remain still, and concentrate on a focal point on the screen in front of them so that less myogenic and ocular artifacts occur. Also, under resting conditions, there are less variations in the head impedance [23], which is important for data acquisition in this study.

### 2.2. Simulated Data

**Figure 2:**
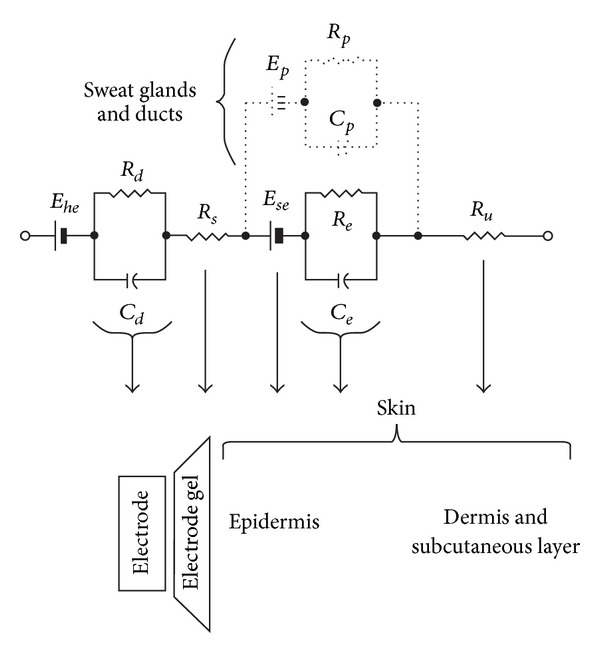
Electrical equivalent circuit for the electrode-skin interface and the underlying skin [24].

 To quantitatively assess and optimize the performance of the proposed method and compare the accuracy of different methods in removing the GVS artifacts from the EEG recordings, we used simulated data. The simulation study was carried out by combining the clean (artifact free) EEG recordings with the simulated GVS contamination. To simulate the actual process of the GVS contamination, we paid attention to the physical structure of the electrode-skin interface and the electrical impedance of the head between the points that the EEG and the GVS electrodes are placed. As the skull impedance is much higher than scalp impedance [23], we can assume that the GVS current mainly distributes through the scalp. The skin and the electrode-skin interface can be modeled using a resistive-capacitive circuit [24], as shown in Figure 2.

 In this electrical equivalent circuit, *E*_*he*_ is the half cell potential of the electrode/gel interface, and the parallel combination of resistive *R*_*d*_ and capacitive *C*_*d*_ components represents the impedance associated with the electrode-gel interface. *R*_*s*_ is the series impedance associated with the resistance of the electrode gel. *E*_*se*_ is the potential difference across the epidermis, whose impedance is represented by the resistance *R*_*e*_ and capacitance *C*_*e*_. In general, the dermis and the subcutaneous layer under it behave as an equivalent pure resistance *R*_*u*_. The deeper layers of the skin, containing vascular, nervous components and hair follicles, contribute very less to the electrical skin impedance, but sweat glands and ducts add an equivalent parallel RC network (represented by broken lines in Figure 2) and a potential difference between sweat glands, ducts, dermis, and subcutaneous layers [24]. If we neglect the pure resistance of the deeper layers of skin and the resistance of the electrode gel, we can simplify the impedance structure as follows:

(1) $$\begin{array}{c} Z\left(s\right)\approx \left(\frac{R_{d}}{sR_{d}C_{d}+1}+\frac{R_{e}}{sR_{e}C_{e}+1}||\frac{R_{p}}{sR_{p}C_{p}+1}\right). \end{array}$$

**Figure 3:**
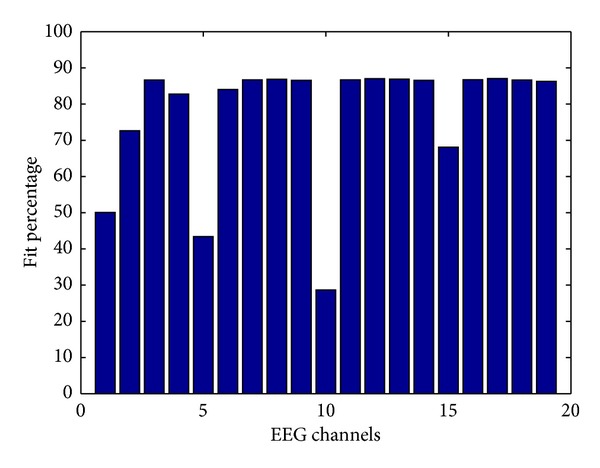
Fit percentage between the simulation output and the measured EEG at each channel.

This equation can be rewritten as

(2) $$\begin{array}{c} Z\left(s\right)\approx \frac{sB_{1}+B_{0}}{s^{2}A_{2}+sA_{1}+1}, \end{array}$$

where *s* is the complex frequency variable, *A*_2_, *A*_1_, *B*_2_, and *B*_1_ represent specific combinations of *R*_*d*_, *R*_*e*_, *R*_*p*_, *C*_*d*_, *C*_*e*_, and *C*_*p*_ for each electrode. This model-based identification approach suggests the following relation between the injected GVS current and the collected EEG voltage at a given electrode:

(3) $$\begin{array}{c} E_{m}=X_{\text{in}}\frac{sB_{1}+B_{0}}{s^{2}A_{2}+sA_{1}+1}+E+W_{\text{noise}}, \end{array}$$

where *E*_*m*_ is the measured EEG, *X*_in_ is the injected GVS current, *E* is the original neural signals or EEG without artifact, and *W*_noise_ is the measurement noise. We simulated this impedance structure to be able to compute the GVS contribution at each EEG channel output:

(4) $$\begin{array}{c} E_{m}^{*}=X_{\text{in}}\frac{sB_{1}+B_{0}}{s^{2}A_{2}+sA_{1}+1}, \end{array}$$

where *E*_m_* represents the GVS artifacts in the measured EEG signals. The simulated impedance structure between GVS electrodes and all 19 EEG electrodes was used to calculate the output voltage due to the GVS current (the GVS artifact) at each EEG electrode (Figure 3).

The fit percentage is a measure of the relative energy fraction in the simulated GVS artifact calculated as given by:

(5) $$\begin{array}{c} \text{fit}=100\left(1-\frac{\sum {\left(E_{m}\left(t\right)-E_{m}^{*}\left(t\right)\right)}^{2}}{\left(\sum \left(E_{m}\left(t\right)-\text{mean}{\left(E_{m}\left(t\right)\right)}^{2}\right)\right)}\right). \end{array}$$

The results show that the fitness of simulated GVS artifact is higher at the EEG electrodes which are closer to the GVS electrodes and it is lower at further channels like channel 15 (Pz), channel 10 (Cz), channel 5 (Fz), channel 1 (FP1), and channel 2 (FP2). According to (2), we can assume that the skin impedance model is a low-order, continuous-time transfer function with one zero and two poles. To simulate the skin impedance structure, we used an iterative nonlinear least-squares algorithm to minimize a selected cost function taken as the weighted sum of the squares of the errors. This algorithm has been applied to real measured data, and the parameters of the impedance model were identified for each EEG electrode. For instance, the simulated electrical equivalent impedance for channel 18 (O1, occipital) has been calculated as:

(6) $$\begin{array}{c} Z\left(s\right)=K_{p}\frac{1+sT_{z}}{s^{2}T_{w}^{2}+2s\zeta \cdot T_{w}+1} \end{array}$$

with *K*_*p*_ = −40921, *T*_*w*_ = 0.10848, *ζ* = 4.7863, and *T*_*z*_ = −2.3726. We used this modeled impedance to simulate the output signal due to scalp propagation between channel 18 and the GVS electrodes (the simulated GVS artifact) which is the dominant term of the total measured EEG signals, with a high fit percentage of about 87%.

**Figure 4:**
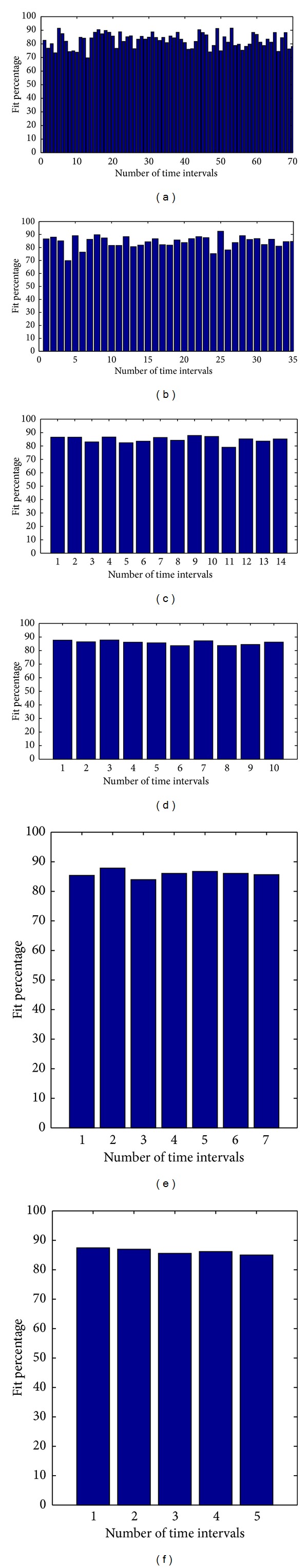
The fit percentage for the simulated GVS artifact at channel 18 for time intervals (a) 1 sec, (b) 2 sec, (c) 5 sec, (d) 7 sec, (e) 10 sec, and (f) 14 sec.

We calculated the impedance models using the entire EEG data collected in each trial (70 seconds). To address the concern about the time-variant properties of the scalp impedance, we computed the impedance models for shorter time intervals (e.g., 1s, 2s, 5s, 7s, 10s, and 14s) and analyzed the fitness of the simulated GVS artifact with the measured EEG data (Figure 4).

The results show that the fitness of the models does not vary for different lengths of time intervals, and for different time intervals it is very close to the fitness of the output model using the entire 70 seconds EEG data, which is around 87%. The above results indicate that the impedance of the scalp can be represented by one transfer function for the entire trial. To simulate the measured EEG data during the GVS, we combined the simulated GVS artifacts with the clean EEG data collected right before the GVS is applied, in order to get a global data set with known EEG and GVS artifact components. This facilitates a quantitative comparison of the effectiveness of the method in removing the undesirable artifact signals.

### 2.3. Regression-Based Methods for Artifact Removal

 The injected GVS current and the EEG signals are recorded concurrently by the measurement system, while the GVS current distribution through the scalp contaminates the recorded EEG signals. We can use the recorded GVS current as a reference to identify the GVS artifacts in the measured EEG signals. To identify the GVS artifacts in the contaminated EEG signals, we applied time-series regression methods using different model structures. One class of model structures is the *discrete-time polynomial* models, described by the following general equation:

(7) $$\begin{array}{c} A\left(q\right)y\left(t\right)=\frac{B\left(q\right)}{F\left(q\right)}u\left(t\right)+\frac{C\left(q\right)}{D\left(q\right)}e\left(t\right). \end{array}$$

Here *u*(*t*) is the recorded GVS current, *y*(*t*) is the estimated GVS artifact, and *e*(*t*) is a white noise (mean = 0, variance = *σ* ^2^) which represents the stochastic part of the model. *A*(*q*), *B*(*q*), *C*(*q*), *D*(*q*), and *F*(*q*) are polynomials in terms of the time-shift operator q which describe the influence of the GVS current and measurement noise on the EEG data. Model structures such as ARMAX, Box-Jenkins, and Output-Error (OE) are the subsets of the above general polynomial equation. In ARMAX model *F*(*q*) and *D*(*q*) are equal to 1, in Box-Jenkins *A*(*q*) is equal to 1, and in Output-Error model *A*(*q*), *C*(*q*), and *D*(*q*) are equal to 1.

Another class of model structures is Hammerstein-Wiener model, which uses one or two static nonlinear blocks in series with a linear block. This model structure can be employed to capture some of the nonlinear behavior of the system. The linear block is a discrete transfer function, represents the dynamic component of the model, and will be parameterized using an Output-Error model similar to the previous model. The nonlinear block can be a nonlinear function such as dead-zone, saturation, or piecewise-linear functions. As we have not observed any dead-zone or saturation type of nonlinearity in our data, we chose the piecewise-linear function by which we can break down a nonlinear system into a number of linear systems between the breakpoints.

We also used *state-space* models in which the relation between the GVS signals, noise, and the GVS artifacts are described by a system of first-order differential equations relating functions of the state variables, noise, and the GVS signal to the first derivatives of the state variables and Output equations relating the state variables and the GVS signal to the GVS artifact.

### 2.4. Adaptive Filtering Methods for Artifact Removal

 Adaptive filtering is another approach to remove artifacts. This method is specifically suitable for real time applications. The adaptive filter uses the received input data point to refine its properties (e.g., transfer function or filter coefficients) and match the changing parameters at every time instant. These filters have been employed to remove different EEG artifacts [25].

In our application, the primary input to the adaptive filter system is the measured contaminated EEG signal *E*_*m*_(*n*) as a mixture of a true EEG *E*_*t*_(*n*) and an artifact component *z*(*n*). The adaptive filter block takes the GVS current *i*_GVS_(*n*) as the reference input and estimates the artifact component. The filter coefficients *h*_*m*_ are adjusted recursively in an optimization algorithm driven by an error signal:

(8) $$\begin{array}{c} e\left(n\right)=E_{m}\left(n\right)-{\hat{E}}_{\text{GVS}}\left(n\right)=E_{t}\left(n\right)-\left[z\left(n\right)-{\hat{E}}_{\text{GVS}}\left(n\right)\right], \end{array}$$

where

(9) $$\begin{array}{c} {\hat{E}}_{\text{GVS}}\left(n\right)=\sum_{m=1}^{M}h_{m}\cdot i_{\text{GVS}}\left(n+1-m\right). \end{array}$$

Because of the function of vestibular system which modulates the stimulation signals [26], there is no direct linear correlation between the true EEG *E*(*n*) and the GVS current *i*_GVS_(*n*). On the other hand, there is a strong correlation between the GVS artifact *z*(*n*) and *i*_GVS_(*n*), so we can calculate the expected value of *e* ^2^ as follows:

(10) $$\begin{array}{c} E\left[e^{2}\left(n\right)\right]=E\left[{\left(E_{m}\left(n\right)-{\hat{E}}_{\text{GVS}}\left(n\right)\right)}^{2}\right] \end{array}$$

or

(11) $$\begin{array}{c} E\left[e^{2}\left(n\right)\right]=E\left[E_{t}^{2}\left(n\right)\right]-E\left[{\left(z\left(n\right)-{\hat{E}}_{\text{GVS}}\left(n\right)\right)}^{2}\right]. \end{array}$$

And as the adjustment of the filter coefficients does not affect the *E*[*E*_*t*_^2^(*n*)], therefore minimizing the term $E[(z(n)-{\hat{E}}_{\text{GVS}}{\left(n)\right)}^{2}]$ is equivalent to minimizing *E*[*e* ^2^(*n*)].

Among the various optimization techniques, we chose the Recursive Least-Squares (RLS) and the Least Mean Squares (LMS) for our application. In the section “Comparison of the performance of different artifact removal methods”, we compared the results of adaptive filters with those of the other methods.

### 2.5. Wavelet Decomposition Methods

**Figure 5:**
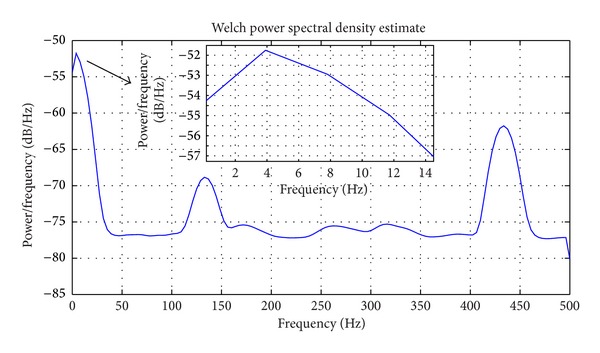
The GVS current power spectrum.

 In this section, we explain how we employ the wavelet methods to enhance the performance of our artifact removal method. The applied GVS current in this study is a pink noise with frequency band of 0.1–10 Hz. Both the GVS current and the EEG data are acquired at the sampling rate of 1000 Hz. After antialiasing filtering, the signals are in a frequency range of 0–500 Hz. The following is the power spectrum of the GVS current using *Welch's method* (Figure 5).

 As shown above, the main GVS frequency components are in the range of 0.1 to 10 Hz. To achieve the best fit between the estimated GVS contribution and the measured EEG at each EEG channel, we broke down the recorded GVS current and the contaminated EEG data into various frequency bands by means of wavelet analysis and estimated the GVS artifacts in each frequency band. Wavelet transform is able to construct a high resolution time-frequency representation of nonstationary signals like EEG signals. In wavelet transform, the signal is decomposed into a set of basis functions, obtained by dilations and shifts of a unique function *ψ* called the *mother* or the *prototype* wavelet, as opposed to a sine wave which is used as the basis function in the Fourier Transform. When the signals are discrete, the *discrete wavelet transform* (DWT) algorithm can be applied, and the set of basis functions are defined on a *“dyadic”* grid in the time-scale plane as

(12) $$\begin{array}{c} \psi_{j,k}\left(t\right)=2^{-(j/2)}\psi \left(2^{-j}t-k\right), \end{array}$$

where 2^*j*^ governs the amount of scaling and *k*2^*j*^ governs the amount of translation or time shifting. The wavelet transform is the inner product of the basis wavelet functions and the signal in the time domain. In the DWT algorithm, the discrete time-domain signal is decomposed into high frequency or details components and low frequency or approximation components through successive low pass and high pass filters. For multi resolution analysis, the original signal is decomposed into an approximation and details parts. The approximation part is decomposed again by iterating this process; thus one signal can be decomposed into many components. The basic DWT algorithm does not preserve translation invariance. Consequently a translation of wavelet coefficients does not necessarily correspond to the same translation of the original signal. This nonstationary property originates from the downsampling operations in the pyramidal algorithm. The algorithm can be modified by inserting 2^*j*^ − 1 zeros between filters coefficients of the layer *j*, instead of down-sampling. This modified version of the DWT algorithm is called *stationary wavelet transform* (SWT), and it can preserve the translation invariance property. In this study, we applied both DWT and SWT, to decompose the EEG signals using different mother wavelets such as *Symlet* and *Daubechies* of different orders. Both the GVS current and the simulated EEG signals were decomposed into 12 levels, and thus we have the frequency bands for approximation and detail components, shown in Table 2.

### 2.6. ICA-Based Methods for Artifact Removal

**Figure 6:**
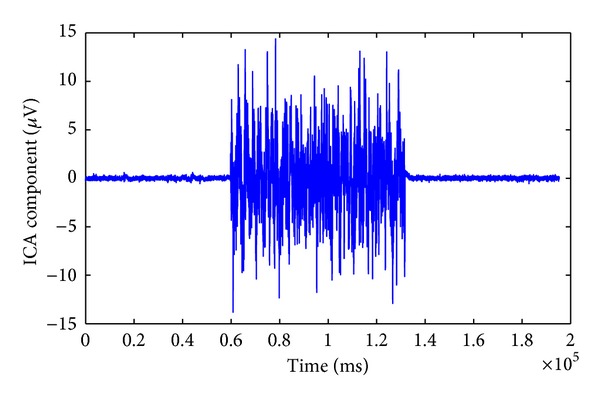
The ICA component attributed to the stimulus artifact, 72 seconds in the middle.

*Independent Component Analysis* (ICA) is a statistical method used to extract independent components from a set of measured signals. This method is a special case of the *Blind Source Separation* methods, where the *K* channels of the recorded EEG signals (*E*(*t*) = *e*_1_(*t*),…, *e*_*K*_(*t*)) are assumed to be a linear combination of *N*(*N* ≤ *K*) unknown independent sources (*S*(*t*) = *s*_1_(*t*),…, *s*_*N*_(*t*)):

(13) $$\begin{array}{c} E\left(t\right)=MS\left(t\right), \end{array}$$

where *M* is the unknown mixing matrix defining weights for each source contributions to the EEG signals recorded at each channel. In ICA, the measured *K* channel EEG signals are taken into an *N* dimensional space and projected onto a coordinate frame where the data projections are minimally overlapped and maximally independent of each other. There are various algorithms with different approaches to find the independent components, such as minimizing the mutual information or maximizing the joint entropy among the data projections. The ICA algorithm we used in this study is the *extended Infomax* algorithm [27] which is a modified version of the Infomax algorithm proposed by Bell and Sejnowski [28]. It uses a learning rule that switches between different types of distributions such as Sub-gaussian and Super-gaussian sources. The extended Infomax algorithm is implemented in EEGLAB MATLAB toolbox [29] and widely used to analyze EEG studies. The ICA was applied to the measured EEG set to find the GVS artifacts components. To remove the GVS artifact we need to find all components that are attributed to the GVS applied to the subject. These components can be identified by calculating the correlation coefficient between the ICA components and the GVS signal. The temporal structure of the GVS artifact components is also different from the other components as, during the time that the GVS is applied, a large amplitude artifact appears (Figure 6).

**Figure 7:**
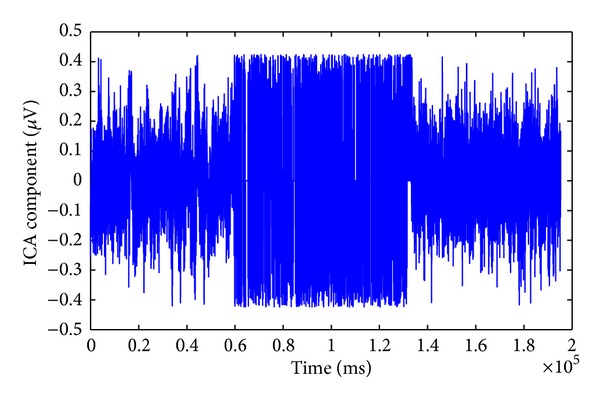
The ICA component attributed to the stimulus artifact after applying the threshold.

We tried two approaches to remove the artifact. The first approach is to zero out the artifact signals from the components that account for the GVS parasitic influence and obtain a new cleaned-up source matrix $\hat{S}(t)$. The second approach is to apply a threshold on the artifact components, in order to extract the artifact spikes and set them to zero. The threshold was set at three standard deviations above the mean of the EEG signal without the artifact (e.g., the signal before applying the GVS), and all data points with amplitude over the threshold were set to zero. Thus we obtained a new source matrix, $\hat{S}(t)$, with the modified components. The threshold at 3 standard deviations of the original neural signals enables us to keep a major part of the original neural activities untouched as much as possible (Figure 7). 

Eventually, we reconstruct ICA-corrected EEG signals as:

(14) $$\begin{array}{c} \hat{E}\left(t\right)=M\hat{S}\left(t\right), \end{array}$$

where $\hat{E}(t)$ is the new data set which represents the estimated artifact-free data.

### 2.7. The Proposed Artifact Removal Method

**Figure 8:**
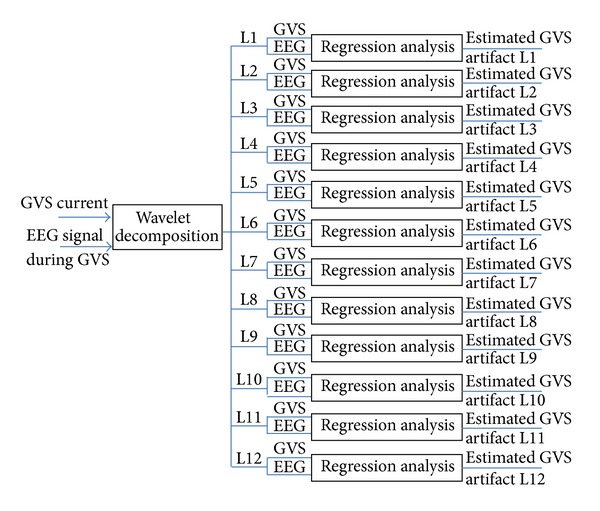
Flowchart of the process for detecting GVS artifacts in the proposed method.

In the proposed method, we decomposed the EEG and GVS current signals in 12 frequency bands (Table 2), and then using the regression methods, we estimated the GVS artifact components in each frequency band.  Figure 8 shows the process for detecting GVS artifacts. As shown in this flowchart, in each frequency band, the GVS artifacts are detected through a regression analysis, where the GVS signals are taken as the reference signals.

The estimated GVS artifact frequency components are subtracted from the contaminated EEG frequency components. The wavelet decomposition enables us to focus on the frequency bands of interest and calculate the estimated GVS artifacts in each frequency band independently; thus the regression method can deal better with some nonlinear behaviors of the skin in the frequency domain. This wavelet-based time-frequency analysis approach enhances the performance of the artifact removal method. The cleaned-up signal is reconstructed from the proper frequency components of the estimated GVS signal components in the frequency range of interest (e.g., 1 Hz to 32 Hz). We calculated the correlation coefficients between the GVS signals and the estimated GVS artifacts reconstructed from different frequency bands, and we observed that the regression results improve when we reconstruct the estimated GVS artifact components from corresponding frequency bands separately.

The result of the correlation analysis is tabulated in Table 3. In this analysis, the real data from channel O1, occipital EEG, was decomposed into 12 frequency bands, using the SWT algorithm with the mother wavelet db3, and the GVS current was estimated using OE regression model of order 2. We calculated *Pearson's correlation* for the correlation analysis as

(15) $$\begin{array}{c} \text{Corr}\left(u,\hat{y}\right)=\frac{\text{Cov}\left(u,\hat{y}\right)}{\sigma_{u}\cdot \sigma_{\hat{y}}}, \end{array}$$

where *u*(*t*) is the recorded GVS current and ${\hat{y}}_{i}(t)$ is the estimated GVS artifact reconstructed from different frequency components.

 The result shows that the correlation between the GVS signal and the estimated GVS artifact significantly increases by using wavelet decomposition method. We applied the wavelet transform to remove frequency components lower than 0.98 Hz and higher than 31.25 Hz, which are not of the main interest, and the correlation between the GVS signal and estimated GVS artifact was increased up to 0.9899.

We employed both SWT and DWT algorithms in the proposed artifact removal method. The difference between SWT and DWT algorithms was briefly explained in the wavelet analysis section. We also used various regression models to estimate the GVS artifact. To assess the performance of the proposed method using different algorithms and models, we applied our method to the simulated data and examined the cleaned-up EEG signals in comparison with the original artifact-free EEG signals. For this assessment, not only did we calculate the correlation between the artifact-removed EEG signals and the original artifact-free EEG signals, but also we measured the fitness of the artifact-removed signals based on the *normalized residual sum of squares* which is sometime introduced as the *normalized quadratic error* defined by

(16) $$\begin{array}{c} \text{RS}{\text{S}}_{N}=\frac{\sum {\left(E_{o}\left(t\right)-{\hat{E}}_{o}\left(t\right)\right)}^{2}}{\sum {\left(E_{o}\left(t\right)-\text{mean}\left(E_{o}\left(t\right)\right)\right)}^{2}}, \end{array}$$

where *E*_*o*_(*t*) represents the original artifact-free signal and ${\hat{E}}_{o}(t)$ is the artifact-removed signal.

We measured the performance of the proposed method based on the correlation (15) and the normalized residual sum of squares (16). The choice for the wavelet algorithm and mother wavelet was made such that the performance of the artifact removal method is maximized. To compare different wavelet algorithms and mother wavelets, we employed a number of mother wavelets from two different wavelet families which have been commonly used in EEG signal processing, *Daubechies* (*db*3, *db*4, and *db*5) and *Symlets* (*sym*3, *sym*4, and *sym*5). Both SWT and DWT were used with these mother wavelets in the proposed artifact removal method and applied to the simulated data. We tabulated the normalized residual sum of squares and the correlation between the artifact-removed signals and the original artifact-free signals in the frequency range lower than 31.25 Hz (Table 4).

The results show that SWT algorithm has a superior performance compared to DWT algorithm, and between different mother wavelets both Daubechies and Symlet wavelets with order of 4 performed better than the others.

Another step to improve the performance of the method, is finding an optimum regression method to calculate the estimated GVS artifacts as accurate as possible. We used three different classes of model structure, Output-Error (OE) as a simple special case of the general polynomial model, Hammerstein-Wiener with the piecewise-linear function, and Space-State models, which were all introduced in the “Regression-based approach” section. We employed these models with different orders in the proposed artifact removal method and applied the proposed method using each of these models to the simulated data. In order to compare the performance, we used SWT with Daubechies 4 to decompose the contaminated signals, estimated the GVS artifact using different models, and then assessed the performance in terms of the correlation and the normalized residual sum of squares between the original artifact-free signal and the artifact-removed signal reconstructed in the frequency range lower than 31.25 Hz. The results are tabulated in Table 5.

 For nonlinear Hammerstein-Wiener models we used the piecewise-linear function and broke down the EEG signal into a number of intervals. We tried a various number of intervals and observed that, with 4 intervals (or less), we could get the highest correlation and the least residual.

The results show that, between all those models, both Output-Error and nonlinear Hammerstein-Wiener have better performance. We employed these regression models to maximize the performance of the proposed method, then we applied the proposed method to the real data.

We also used two ICA-based methods for removing the artifact: filtering out the artifact components and applying a threshold on the artifact components amplitude to remove the artifact spikes beyond the threshold.

To assess the performances of the ICA methods on the simulated data, we calculated both the correlation and the normalized residual sum of squares between the artifact-removed EEG signals and the original artifact-free EEG signals.

We compared the ICA-based methods with the proposed methods using the Output-Error and nonlinear Hammerstein-Wiener models order 2, along with 12-level STW decomposition with DB4 mother wavelet (Tables 6 and 7).

### 2.8. Comparison of Different Artifact Removal Methods

 We applied different artifact removal methods on real EEG data acquired during application of GVS. We used the data from channel O1 (occipital EEG) of different subjects in EEG/GVS studies. We applied stimulation signals of different amplitudes in our experiments and observed consistent results from these experiments. By calculating the correlation coefficients between the GVS signals and the estimated GVS artifacts, we compared the performance of these methods. First we compare ICA-based, regression-based, and adaptive filters without using the wavelet analysis. Then we use the proposed method where the wavelet analysis was employed to improve the performance of our artifact removal method.

The best algorithms for ICA-based methods, best models for regression-based methods and best filters for adaptive filtering methods were selected. Between different ICA algorithms (as mentioned in the section “ICA-based artifact removal methods”), the extended Infomax showed better results. Between regression-based methods (as previously introduced in the section “Regression-based artifact removal methods”), OE order 2 showed better performance, and between adaptive filters (as previously introduced in the section “Adaptive filtering methods for artifact removal”), RLS filter with the forgetting factor of 0.99997, the filter length of 2, LMS filter with the adaptation gain of 0.5, and the filter length of 3 had better performance. We tabulated (Table 8) the correlation between the GVS signals and the estimated GVS artifacts.

The results show that, between all the above methods, the regression-based methods are able to estimate the GVS artifacts with higher correlation with the original GVS signals. Thus, we employed the regression-based method along with the wavelet analysis in our proposed method to achieve the best performance in removing GVS artifact. The wavelet decomposition method improves the estimation of the GVS artifacts in both correlation performance and robustness. This is due to the separate transfer function estimations for each frequency band, aspect that makes it less prone to nonlinear skin behavior or to other noise sources. Furthermore, with wavelet decomposition, we can filter out the frequency components that are not of interest. Removing those frequency components can improve the results of the regression analysis as well. The cleaned EEG data is reconstructed from the frequency range of interest (e.g., 1 Hz to 32 Hz).

Using a correlation analysis, we show how the wavelet-based time-frequency analysis approach enhances the performance of the artifact removal method. We calculated the correlation coefficients between the GVS signals and the estimated GVS artifacts reconstructed from different frequency bands (tabulated in Table 9). We observed that by focusing on the frequency components of interest, for example, between 1 Hz to 32 Hz, we could achieve much higher correlation between the estimated and original GVS signals.

As shown in Table 9, after removing the frequency bands lower than 0.98 Hz and larger than 31.25 Hz, which were outside our interest at the present time, the correlation between the GVS signal and the estimated GVS artifact significantly increases from 0.7673 to 0.9899 by using wavelet decomposition method.

**Figure 9:**
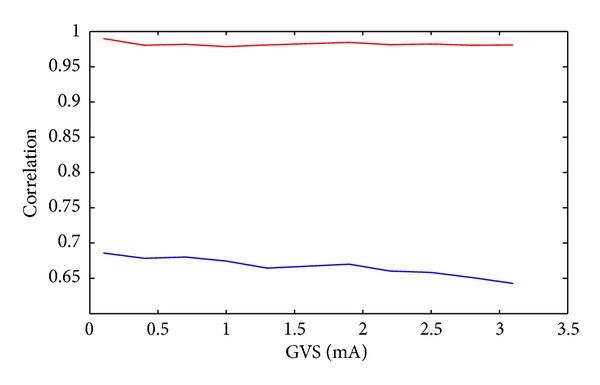
Correlation between the GVS signal and the estimated GVS artifact using the proposed method (red) and the ICA method (blue) for different GVS amplitudes.

So far, we showed the proposed method has superior performance than the other methods when it is applied to low-amplitude stochastic GVS signals up to 1 mA. We also applied our artifact removal method to EEG/GVS data sets collected by our other collaborator in the Sensorimotor Physiology Laboratory, where higher amplitude pink noise GVS up to 3100 *μ*A was applied in the EEG/GVS studies. In one data sets, pink noise GVS in a wide range of amplitudes from 100 *μ*A to 3100 *μ*A (each 300 *μ*A) was applied, and the EEG/GVS data were collected. We compared the performance of the proposed method and the extended Infomax ICA method. The results show that while the performance of the ICA method deteriorates as the GVS amplitude is increased, the proposed method provides a robust performance (Figure 9).

## 3. Results

 In the section “The proposed artifact removal method”, we optimized the proposed method using the simulated data. To find the optimum algorithms for signal decomposition, we compared the SWT and DWT decomposition algorithms using different mother wavelets (the results shown in Table 4), and to achieve better estimation of the GVS artifacts, we employed different model structures (results shown in Table 5).

In the optimized algorithm, we employed the SWT decomposition algorithm using DB4 mother wavelet and decomposed the signals into 12 frequency bands. This enabled us to separate the GVS artifact into different frequency bands and estimate the artifact using a time-domain regression model. The comparison of the different model structures shows that the Output-Error (OE) and the nonlinear Hammerstein-Wiener order 2 have similar performances, better than the other models.

In the previous section, we compared the performance of different methods and observed that how the combining of wavelet decomposition and regression analysis (Table 9) can improve the performance of the artifact removal method for GVS/EEG studies.

**Figure 10:**
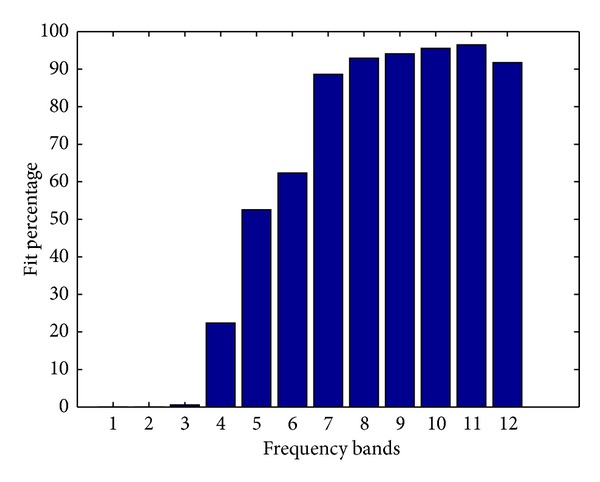
The fit percentage of the detail components of the estimated GVS artifacts using the OE model order 2 in each frequency band.

**Figure 11:**
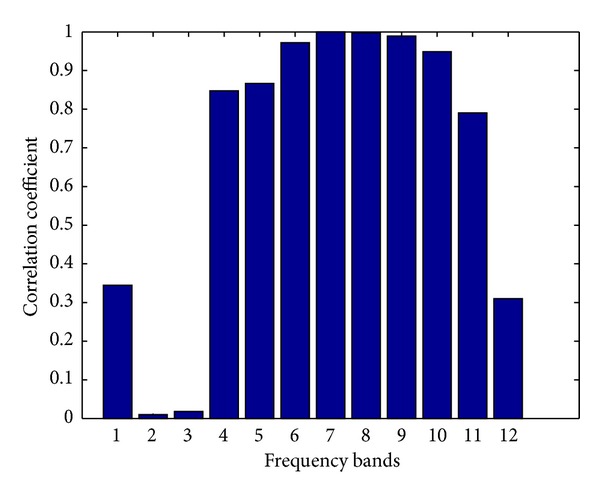
The correlation between the detail components of the estimated GVS signals and the GVS signals for the simulated data using the OE model order 2 in each frequency bands.

Using the proposed method, we can focus on specific frequency bands and remove the GVS artifact with better performance in each frequency band, separately. Figures 10 and 11 show the fit percentage (5) and the correlation (15) between the detail components of the estimated GVS signals and the GVS signals for the simulated data in the frequency bands introduced in Table 2.

**Figure 12:**
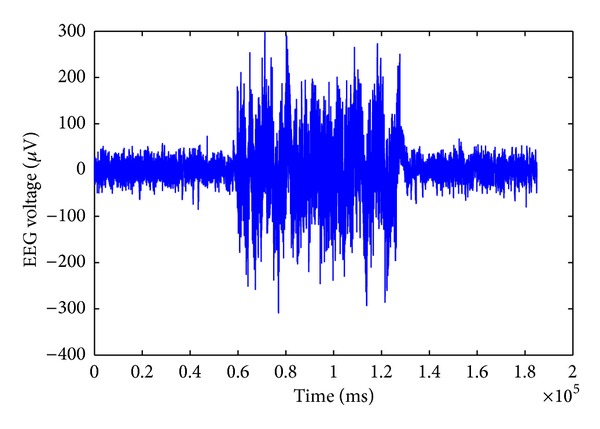
The occipital EEG channel data after applying the proposed artifact removal method using the frequency components lower than 64 Hz.

**Figure 13:**
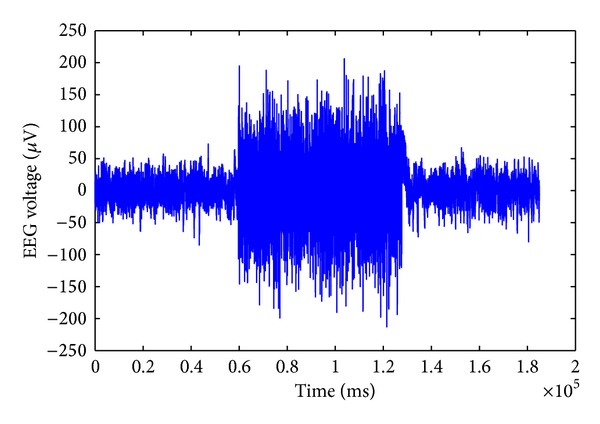
The occipital EEG channel data after applying the proposed artifact removal method using the frequency components between 1 Hz to 32 Hz.

**Figure 14:**
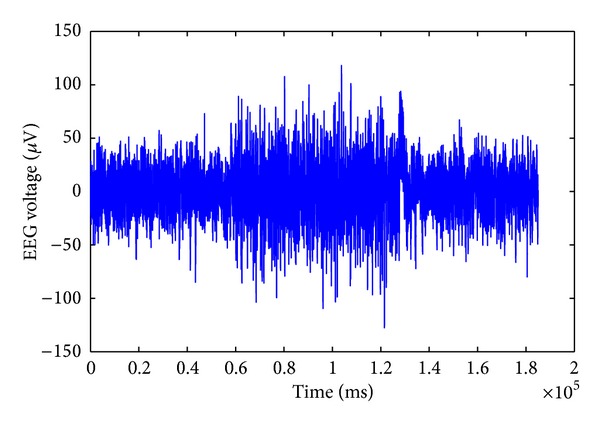
The occipital EEG channel data after applying the proposed artifact removal method using the frequency components between 1 Hz to 16 Hz.

The results show that for frequency components L6 to L10, which correspond approximately to 8–16 Hz, 4–8 Hz, 2–4 Hz, 1-2 Hz, and 0.5–1 Hz bands, we can achieve higher performance in rejecting the GVS artifacts separately. One of the reasons of the robustness of the method is building separate equivalent transfer functions for the GVS signals for each frequency band which helps in maintaining the performance of the algorithms for a large range of GVS intensity levels and frequency ranges. To illustrate the importance of the wavelet analysis, we depicted the artifact-removed signals using different frequency components (Figures 12, 13, and 14).

Figure 14 shows that when we use specific frequency components to estimate the GVS artifacts, we can significantly suppress the GVS artifact and achieve high *signal to artifact ratio* (SAR). SAR is defined as the ratio of the signal amplitude to the artifact amplitude in decibels (dB). We can achieve an SAR of −1.625 dB in the frequency range of 1 Hz–16 Hz, while, using the frequency components in the range of 1 Hz–32 Hz (Figure 13), we can obtain a SAR of −10.498 dB; using the frequency components in the range of 1 Hz–64 Hz (Figure 12), we have an SAR of −13.863 dB. In the original contaminated EEG signals, without removing the GVS artifact (Figure 1), the SAR is −32.189 dB.

## 4. Discussion

 In the section “Simulated data”, we showed that by simulating the skin impedance and estimating the transfer function of the skin (one function for the whole frequency range), we could reconstruct a major portion of the GVS artifact. As an example, for channel 18, around 87% of the GVS artifact was reconstructed (Figure 3), thus we could simulate the contaminated EEG signals to assess the performance of the proposed method.

Using the wavelet decomposition, we were able to reconstruct up to 96% of the GVS artifact components in some frequency bands, especially in the frequency range of the GVS signals (Figure 10).

We showed that the use of the wavelet decomposition can improve the time domain regression approach to estimate the GVS artifacts. By means of the combination of the regression and wavelet analysis in the proposed artifact removal method, we were able to focus on different frequency bands and significantly improve the SAR of the contaminated EEG data in specific frequency bands.

The proposed method and the ICA-based methods behave differently in rejecting the GVS artifact. We observed a high correlation between the estimated GVS artifacts and the original GVS signals using the proposed method, but we could not obtain a good correlation using the ICA-based methods.

As illustrated earlier, we cannot completely remove the GVS contamination in all frequency ranges (e.g., over 16 Hz). Removing the whole GVS artifacts remains a problem for the future approaches.

In this study we also observed that nonlinear Hammerstein-Wiener model of the second order, using piecewise-linear blocks with 4 breakpoints (or less), provided the same performance as the Output-Error model of the second order. This implies that the relationships between the GVS artifacts at the EEG electrodes and the injected GVS current are linear and remain constant over the entire epoch. Our simulation study results also showed that the impedance models between the EEG electrodes and the GVS electrodes remain constant over the entire epoch (Figure 4) and using short epochs would not improve the fitness of the impedance models and the estimation of the GVS artifacts. As a matter of fact, it may even worsen the estimation of time-domain characteristics.

We also showed that, when we apply the proposed method to remove the GVS artifacts, less distortion is introduced in the cleaned EEG signals, compared to the distortion that the other methods (e.g., ICA-based methods) introduce. Furthermore, using the proposed method, we do not need to collect and process all EEG channels as in the ICA-based analysis; therefore it is much faster than the ICA-based methods. This allows us to have a simple experimental setup for collecting EEG signals with less EEG channels for the GVS studies which makes the preparation for the data acquisition session take less time before the subject gets tired, and more myogenic and ocular artifacts are introduced. Compared to the ICA methods, the proposed method is easier to be implemented in a real time system for future applications.

## Figures and Tables

**Table 1:** EEG channels.

ch1	ch2	ch3	ch4	ch5	ch6	ch7	ch8	ch9	ch10
FP1	FP2	F7	F3	Fz	F4	F8	T7	C3	Cz

ch11	ch12	ch13	ch14	ch15	ch16	ch17	ch18	ch19	ch20
C4	T8	P7	P3	Pz	P4	P8	O1	O2	Ref

**Table 2:** Frequency bands for approximation and details components.

	L1	L2	L3	L4	L5	L6
Approximation	0–250	0–125	0–62.5	0–31.25	0–15.75	0–7.87
Details	250–500	125–250	62.5–125	31.25–62.5	15.75–31.25	7.87–15.75

	L7	L8	L9	L10	L11	L12

Approximation	0–3.93	0–1.96	0–0.98	0–0.49	0–0.24	0–0.12
Details	3.93–7.87	1.96–3.93	0.98–1.96	0.49–0.98	0.24–0.49	0.12–0.24

**Table 3:** Correlation between the GVS signal and the estimated GVS artifact reconstructed from different frequency components.

	Estimated GVS artifact without wavelet decomposition	Estimated GVS artifact from 0.12 Hz to 250 Hz	Estimated GVS artifact from 0.24 Hz to 125 Hz	Estimated GVS artifact from 0.49 Hz to 62.5 Hz

Correlation	0.6960	0.8463	0.9168	0.9725

	Estimated GVS artifact from 0.49 Hz to 31.25 Hz	Estimated GVS artifact from 0.49 Hz to 15.75 Hz	Estimated GVS artifact from 0.98 Hz to 31.25 Hz	Estimated GVS artifact from 0.98 Hz to 15.75 Hz

Correlation	0.9776	0.9769	0.9899	0.9899

**Table 4:** Correlation and normalized residual sum of squares between the artifact-removed signals and the original artifact-free EEG signals for simulated data using different wavelet decomposition algorithms.

	DWT db3	DWT db4	DWT db5	DWT db6	DWT sym3	DWT sym4	DWT sym5	DWT sym6
Corr.	0.8781	0.9023	0.9155	0.9242	0.8781	0.9023	0.9156	0.9242
RSS_*N*_	0.5517	0.4870	0.4503	0.4255	0.5517	0.4870	0.4503	0.4255

	SWT db3	SWT db4	SWT db5	SWT db6	SWT sym3	SWT sym4	SWT sym5	SWT sym6

Corr.	0.9932	0.9933	0.9933	0.9932	0.9932	0.9933	0.9933	0.9932
RSS_*N*_	0.1710	0.1700	0.1705	0.1714	0.1710	0.1700	0.1705	0.1714

**Table 5:** Correlation and normalized residual sum of squares between the artifact-removed signals and the original artifact-free EEG signals for simulated data using different models for estimating the GVS artifacts.

	OE2	OE3	OE4	OE5	NLHW2
Corr.	0.9933	0.9933	0.9933	0.9822	0.9934
RSS_*N*_	0.1700	0.1701	0.1704	0.2267	0.1711

	SS2	SS3	SS4	NLHW3	NLHW4

Corr.	0.9933	0.8105	0.7466	0.9926	0.9851
RSS_*N*_	0.1704	0.7628	0.9174	0.1230	0.1725

**Table 6:** Correlation and normalized residual sum of squares between the artifact-removed signals and the original artifact-free EEG signals for simulated data using the proposed method and ICA-based methods.

	Removing the ICA artifact component	Applying threshold to the ICA artifact component	SWT decomposition with DB4 modeled with OE2	SWT decomposition with DB4 modeled with NLHW2
Corr.	0.6445	0.6171	0.9933	0.9934
RSS_*N*_	0.9567	1.0241	0.1700	0.1711

**Table 7:** Correlation between the GVS signals and the estimated GVS artifact extracted from EEG signals for real data using the proposed method and ICA-based methods.

	Removing the ICA artifact component	Applying threshold to the ICA artifact component	SWT decomposition with DB4 modeled with OE2	SWT decomposition with DB4 modeled with NLHW2
Corr.	0.6859	0.6858	0.8743	0.8743

**Table 8:** Correlation between the GVS signals and the estimated GVS artifact extracted from EEG signals for real data using different methods.

Method	Correlation
ICA-Infomax method (remove the artifact component)	0.6859
ICA-Infomax method (threshold the artifact component)	0.6858
Regression method with OE2	0.7673
RLS Adaptive filter (forgetting factor: 0.99997, length: 2)	0.7615
LMS Adaptive filter (adaptation gain: 0.5, length: 3)	0.7010

**Table 9:** Correlation between the GVS signal and the estimated GVS artifact reconstructed from different frequency components for real data.

Frequency band	Correlation
Estimated GVS artifact without wavelet decomposition	0.7673
Estimated GVS artifact from 0.12 Hz to 250 Hz	0.8463
Estimated GVS artifact from 0.24 Hz to 125 Hz	0.9168
Estimated GVS artifact from 0.49 Hz to 62.5 Hz	0.9725
Estimated GVS artifact from 0.49 Hz to 31.25 Hz	0.9776
Estimated GVS artifact from 0.49 Hz to 15.75 Hz	0.9769
Estimated GVS artifact from 0.98 Hz to 31.25 Hz	0.9899
Estimated GVS artifact from 0.98 Hz to 15.75 Hz	0.9899

## References

[1] Yamamoto Y, Struzik ZR, Soma R, Ohashi K, Kwak S (2005). Noisy vestibular stimulation improves autonomic and motor responsiveness in central neurodegenerative disorders. *Annals of Neurology*.

[2] Pan W, Soma R, Kwak S, Yamamoto Y (2008). Improvement of motor functions by noisy vestibular stimulation in central neurodegenerative disorders. *Journal of Neurology*.

[3] Pal S, Rosengren SM, Colebatch JG (2009). Stochastic galvanic vestibular stimulation produces a small reduction in sway in parkinson’s disease. *Journal of Vestibular Research*.

[4] Yamamoto Y, Soma R, Struzik ZR, Kwak S Can electrical vestibular noise be used for the treatment of brain diseases?.

[5] Utz KS, Dimova V, Oppenlander K, Kerkhoff G (2010). Electrified minds: transcranial direct current stimulation (tdcs) and galvanic vestibular stimulation (gvs) as methods of non-invasive brain stimulation in neuropsychology—a review of current data and future implications. *Neuropsychologia*.

[6] Shackman AJ, McMenamin BW, Slagter HA, Maxwell JS, Greischar LL, Davidson RJ (2009). Electromyogenic artifacts and electroencephalographic inferences. *Brain Topography*.

[7] McMenamin BW, Shackman AJ, Maxwell JS (2010). Validation of ica-based myogenic artifact correction for scalp and source-localized EEG. *NeuroImage*.

[8] Crespo-Garcia M, Atienza M, Cantero JL (2008). Muscle artifact removal from human sleep EEG by using independent component analysis. *Annals of Biomedical Engineering*.

[9] McMenamin BW, Shackman AJ, Maxwell JS, Greischar LL, Davidson RJ (2009). Validation of regression-based myogenic correction techniques for scalp and source-localized EEG. *Psychophysiology*.

[10] Gao J, Yang Y, Lin P, Wang P, Zheng C (2010). Automatic removal of eye-movement and blink artifacts from EEG signals. *Brain Topography*.

[11] Schlogl A, Keinrath C, Zimmermann D, Scherer R, Leeb R, Pfurtscheller G (2007). A fully automated correction method of eog artifacts in eeg recordings. *Clinical Neurophysiology*.

[12] Magjarevic R, Klados MA, Papadelis C, Lithari CD, Bamidis PD, Sloten J, Verdonck P, Nyssen M, Haueisen J The removal of ocular artifacts from eeg signals: a comparison of performances for different methods.

[13] He P, Wilson G, Russell C, Gerschutz M (2007). Removal of ocular artifacts from the EEG: a comparison between time-domain regression method and adaptive filtering method using simulated data. *Medical and Biological Engineering and Computing*.

[14] Schloegl A, Ziehe A, Müller KR Automated ocular artifact removal: comparing regression and component-based methods.

[15] Wallstrom GL, Kass RE, Miller A, Cohn JF, Fox NA (2004). Automatic correction of ocular artifacts in the eeg: a comparison of regression-based and component-based methods. *International Journal of Psychophysiology*.

[16] Grouiller F, Vercueil L, Krainik A, Segebarth C, Kahane P, David O (2007). A comparative study of different artefact removal algorithms for eeg signals acquired during functional MRI. *NeuroImage*.

[17] Erez Y, Tischler H, Moran A, Bar-Gad I (2010). Generalized framework for stimulus artifact removal. *Journal of Neuroscience Methods*.

[18] Morbidi F, Garulli A, Prattichizzo D, Rizzo C, Rossi S (2008). Application of Kalman filter to remove TMS-induced artifacts from EEG recordings. *IEEE Transactions on Control Systems Technology*.

[19] Aksenova TI, Nowicki DV, Benabid A-L (2009). Filtering out deep brain stimulation artifacts using a nonlinear oscillatory model. *Neural Computation*.

[20] Hashimoto T, Elder CM, Vitek JL (2002). A template subtraction method for stimulus artifact removal in highfrequency deep brain stimulation. *Journal of Neuroscience Methods*.

[21] Inuso G, La Foresta F, Mammone N, Morabito FC Brain activity investigation by EEG processing: wavelet analysis, kurtosis and Renyi’s entropy for artifact detection.

[22] Inuso G, La Foresta F, Mammone N, Morabito FC Wavelet-ICA methodology for efficient artifact removal from Electroencephalographic recordings.

[23] Tidswell AT, Gibson A, Bayford RH, Holder DS (2001). Electrical impedance tomography of human brain activity with a two-dimensional ring of scalp electrodes. *Physiological Measurement*.

[24] Webster JG (2009). *Medical Instrumentation-Application and Design*.

[25] Garcés Correa A, Laciar E, Patĩo HD, Valentinuzzi ME (2007). Artifact removal from EEG signals using adaptive filters in cascade. *Journal of Physics*.

[26] Fitzpatrick RC, Day BL (2004). Probing the human vestibular system with galvanic stimulation. *Journal of Applied Physiology*.

[27] Lee T-W, Girolami M, Sejnowski TJ (1999). Independent component analysis using an extended infomax algorithm for mixed subgaussian and supergaussian sources. *Neural Computation*.

[28] Bell AJ, Sejnowski TJ (1995). An information-maximization approach to blind separation and blind deconvolution. *Neural Computation*.

[29] Delorme A, Makeig S (2004). Eeglab: an open source toolbox for analysis of single-trial EEG dynamics including independent component analysis. *Journal of Neuroscience Methods*.

